# Atypical cortical neural activity in internet gaming disorder comorbid with autism spectrum disorder during a cue reactivity task: A magnetoencephalography study

**DOI:** 10.1002/pcn5.70312

**Published:** 2026-03-16

**Authors:** Faisal Budisasmita Paturungi Parawansa, Atsunori Sugimoto, Ekachaeryanti Zain, Yukina Nakazawa, Fuuta Sakuma, Kiyohiro Yoshinaga, Muhammad Dwi Wahyu, Hiroyuki Kasahara, Jun Egawa, Hiroshi Shirozu, Atsuhiko Iijima, Shuken Boku

**Affiliations:** ^1^ Department of Psychiatry Niigata University Graduate School of Medical and Dental Sciences Niigata Japan; ^2^ Department of Neurology, Faculty of Medicine Hasanuddin University Makassar Indonesia; ^3^ Department of Community Psychiatric Medicine Niigata University Graduate School of Medical and Dental Sciences Niigata Japan; ^4^ Department of Psychiatry Niigata Psychiatric Center Nagaoka Niigata Japan; ^5^ Human Sciences and Assistive Technology Course, Department of Electrical and Information Engineering Niigata University Graduate School of Science and Technology Niigata Japan; ^6^ Department of Psychiatry Niigata University Medical and Dental Hospital Niigata Japan; ^7^ Functional Neurosurgery Center, Department of Neurosurgery Fukuoka Sanno Hospital Fukuoka Japan; ^8^ Interdisciplinary Program of Biomedical Engineering, Assistive Technology, and Art and Sports Sciences, Faculty of Engineering Niigata University Niigata Japan; ^9^ School of Health Sciences, Faculty of Medicine Niigata University Niigata Japan

**Keywords:** autism spectrum disorder, cue reactivity task, internet gaming disorder, magnetoencephalography, P2m

## Abstract

**Aim:**

Significant, positive correlations between internet gaming disorder (IGD) and autism spectrum disorder (ASD) are known. Individuals with ASD are prone to problematic internet use due to addiction or restricted interests. Here, we examined cortical neural activity in individuals with IGD comorbid with ASD during a gaming‐related cue reactivity task, using magnetoencephalography (MEG).

**Methods:**

MEG was used to record neural activity in right‐handed male participants aged 11–20 years (11 IGD–ASD, 13 healthy controls; intelligence quotient [IQ] ≥ 80). IGD and ASD were diagnosed per DSM‐5‐TR criteria. During MEG recording, participants viewed gaming cues and neutral base stimuli in a cue reactivity task. Source‐level cortical activity was estimated using minimum norm estimation (MNE), and statistical comparisons were performed using two‐tailed nonparametric permutation tests with false discovery rate (FDR) correction.

**Results:**

In the IGD–ASD group, neural activity was significantly elevated at 137 ms in the right fusiform gyrus during gaming cues compared with the base condition (*p* = 0.000039). Between‐group comparisons under cue conditions (200–270 ms) showed higher right frontal activation (*p* = 0.0028) and lower activation in left lateral occipital (*p* = 0.000092), fusiform (*p* = 0.00025), lingual (*p* = 0.0017), and parahippocampal regions (*p* = 0.000049) in the IGD–ASD group compared with controls.

**Conclusion:**

The IGD–ASD group showed increased right frontal activity and decreased left occipital, fusiform, lingual, and parahippocampal activity during exposure to the gaming cue, suggesting atypical visual and cognitive processing mechanisms in this comorbid group. Further studies comparing individuals with ASD and those with IGD–ASD, as well as by examining the neurophysiological characteristics of individuals with ASD who develop or recover from IGD, might clarify the pathology of these populations.

## INTRODUCTION

Internet gaming disorder (IGD) has been recognized as a behavioral addiction^1^ and a growing public health concern in the digital era. The DSM‐5‐TR outlined nine diagnostic criteria, including preoccupation with internet (online) games, withdrawal, tolerance, loss of control, and significant social functional impairment, while classifying IGD as a condition warranting further study.^2^ Neuroimaging evidence continues to strengthen its potential recognition as an independent psychiatric disorder. The global prevalence of IGD is estimated at approximately 4.7%,^3^ with rates among adolescents and young adults ranging from 4.2% to 24.0%.^4^ In Japan, the prevalence rose from prepandemic levels to 4.1% overall and 8.6% among youth during the COVID‐19 pandemic.^5^ Excessive gaming in childhood and adolescence, a critical period of brain development, has been linked to structural and functional brain alterations,^6^ as well as psychosocial and behavioral difficulties such as neuroticism, aggression, social inhibition, low self‐esteem, and anxiety.^7^ IGD is also associated with poor academic^8^ and occupational performance, interpersonal conflicts, sleep disturbances,^8^ and reduced face‐to‐face interactions.^9^ Some studies further suggest deficits in intelligence, verbal skills, processing speed, and working memory compared with regular or nongamers.^10^ These findings highlight the need to elucidate the neurobiological mechanisms underlying IGD, particularly in young populations.

Autism spectrum disorder (ASD) is a neurodevelopmental disorder characterized by persistent deficits in social communication and restricted, repetitive patterns of behavior, interests, or activities, arising from both genetic and environmental influences on brain development.^2^, ^11^ Significant positive correlations between ASD and IGD are known, suggesting that individuals with ASD are particularly vulnerable to problematic internet use.^12^ Although internet gaming may provide structured opportunities for social interaction and stress reduction, it also carries risks of excessive use and addiction.^13^ Individuals with ASD exhibit higher rates and severity of gaming disorder symptoms,^14^ and the presence of ASD has been identified as a negative prognostic factor in the course of gaming addiction.^15^ Further clinical studies are still needed to distinguish whether excessive gaming behavior in ASD reflects impaired control characteristic of addiction or inherent autistic traits related to restricted interests.^16^

Neuroimaging studies suggest that IGD shares neurobiological characteristics with substance and behavioral addictions, including hyperactivation of reward‐related circuits and hypoactivation of regions governing impulse control, leading to impaired decision‐making.^17^ Key regions implicated include the fusiform gyrus, inferior temporal gyrus, and dorsolateral prefrontal cortex (DLPFC), which are involved in visual and auditory processing, craving,^18^ and higher cognitive functions.^18^, ^19^ Functional magnetic resonance imaging (fMRI) has been widely used to examine cue‐induced reactivity in IGD, but its limited temporal resolution constrains the detection of rapid neural dynamics.^19^, ^20^ Magnetoencephalography (MEG), with millisecond‐level precision, overcomes this limitation, allowing detailed assessment of cortical responses to gaming cues.^21^ Craving in IGD, often elicited by gaming‐related stimuli, reflects dysregulated emotional and cognitive control involving the DLPFC, inferior frontal gyrus, and dorsal anterior cingulate cortex.^22^ Event‐related potential studies have reported increased late positive potential (LPP) amplitudes to gaming cues in IGD, indicating attentional bias and deficits in cognitive control.^8^ The LPP, peaking over centroparietal regions 300–700 ms after stimulus onset, is considered a reliable marker of these processes.^8^, ^9^

Although IGD and ASD have each been investigated extensively, their neurophysiological interaction in individuals with comorbidity remains largely unknown, particularly at the level of rapid cortical dynamics. Previous cue reactivity studies in IGD^8^, ^9^, ^22^ primarily emphasized reward‐related frontal and limbic circuits, while electrophysiological studies in ASD have highlighted atypical perceptual and connectivity patterns. To the best of our knowledge, no prior study has examined millisecond‐scale cortical response dynamics to gaming cues in individuals with comorbid IGD and ASD. Therefore, the present study was designed to focus on the comorbid IGD–ASD phenotype for which consent for the study was obtained during the course of IGD treatment clinical practice.

MEG, with its high temporal resolution, allows detection of early perceptual and cognitive stages of cue processing that may not be captured in fMRI. This approach is particularly relevant in IGD–ASD comorbidity, where perceptual specialization associated with ASD may interact with addiction‐related mechanisms.

## MATERIALS AND METHODS

### Participants and clinical assessments

We enrolled 24 male participants aged 11–20 years^23^, ^24^ in the present study, comprising 11 individuals in the IGD–ASD group and 13 in the healthy control (HC) group. All participants were right‐handed as assessed by the Edinburgh Handedness Inventory^25^ and had^26^ an alternative intelligence quotient (IQ) of ≥70 on the Japanese Adult Reading Test (JART)^26^ in both groups.

Participants in the IGD–ASD group were enrolled at their first visit to the inpatient or outpatient child psychiatry units of Niigata Prefectural Psychiatric Center. All participants met the diagnostic criteria of IGD and ASD according to DSM‐5‐TR criteria and were clinically assessed by a psychiatrist. IGD is listed in DSM‐5‐TR as a condition warranting further study; participants met DSM‐5‐TR IGD criteria, and behavioral features were consistent with ICD‐11 Gaming Disorder. ASD diagnoses were made by child psychiatrists based on DSM‐5‐TR criteria. Standardized diagnostic tools such as the Autism Diagnostic Observation Schedule (ADOS) or Autism Diagnostic Interview–Revised (ADI‐R) were not available, and this is acknowledged as a limitation. Importantly, all patients were newly diagnosed and had not received any prior pharmacological or psychological intervention for IGD at the time of participation.

Participants in the HC group were recruited through an online recruitment procedure. They did not have a diagnosis of IGD nor ASD according to DSM‐5‐TR criteria, confirmed by the interview done by a psychiatrist. A formal structured diagnostic interview was not administered due to feasibility constraints, and this is acknowledged as a methodological limitation.

We excluded participants with a history of head injury, epilepsy, moderate‐to‐severe intellectual developmental disorders, abnormal EEG findings, or any contraindications to MRI and MEG procedures from both groups.

Clinical assessments of behavior were conducted using the Internet Addiction Test (IAT) and the Child Behavior Checklist (CBCL) in both groups, and gaming hours per week were reported. The IAT is a 20‐item self‐report questionnaire^27^ that measures several facets of behavioral addiction, such as preoccupation, loss of control, and psychological dependence, scored on a five‐point Likert scale (1 = rarely, 2 = occasionally, 3 = frequently, 4 = often, and 5 = always). The IAT score range is 20–100, with higher scores indicating greater severity. We used the Japanese version of the IAT,^28^ for which reliability and validity have been established.^29^ The CBCL consists of 113 questions,^30^ scored on a three‐point Likert scale (0 = absent, 1 = occurs sometimes, and 2 = occurs often). It is composed of eight syndrome scales, two combined scale scores (internalizing symptoms, externalizing symptoms), and a total score. We used the standardized Japanese version of the CBCL.^31^ We provided the CBCL scales characterizing ASD, which is the total of the Withdrawn scale and the PDP scale, that were reported to be better at discriminating children with ASD from typically developing children as a Level 1 screening tool.^32^ The CBCL items for the total score of the Withdrawn and the PDP scales used to reflect the ASD symptoms were 2, 3, 4, 7, 21, 23, 25, 62, 63, 67, 70, 71, 76, 80, 92, and 98.^32^, ^33^

### Cue reactivity task

The cue reactivity task, developed by Sugimoto et al.,^34^ consisted of two types of still‐image sets. The first set of Cue stimuli consisted of 64 still images of game‐related visual cues derived from in‐game screen captures of three popular online games (i.e., *Apex Legends*, *Fortnite*, or *Overwatch*). The second set of base stimuli consisted of 64 neutral images created by rotating each cue image by 180°, flipping it horizontally, and then applying Gaussian filtering. They were presented as blurry, still images that were identical in features, including color, screen brightness, and pixel count, to the Cue‐stimulus images, but participants could not tell what was being displayed. All visual stimuli excluded human faces and textual content. Each visual stimulus was matched in size (1200 × 900 pixels, 96 dpi). This task paradigm was originally developed and validated in previous MEG studies of cue reactivity in IGD,^34^ where it reliably elicited cortical responses associated with attentional bias toward gaming‐related stimuli. The use of visually matched base stimuli allowed control for low‐level visual features while isolating cue‐specific processing.

**Figure 1:**
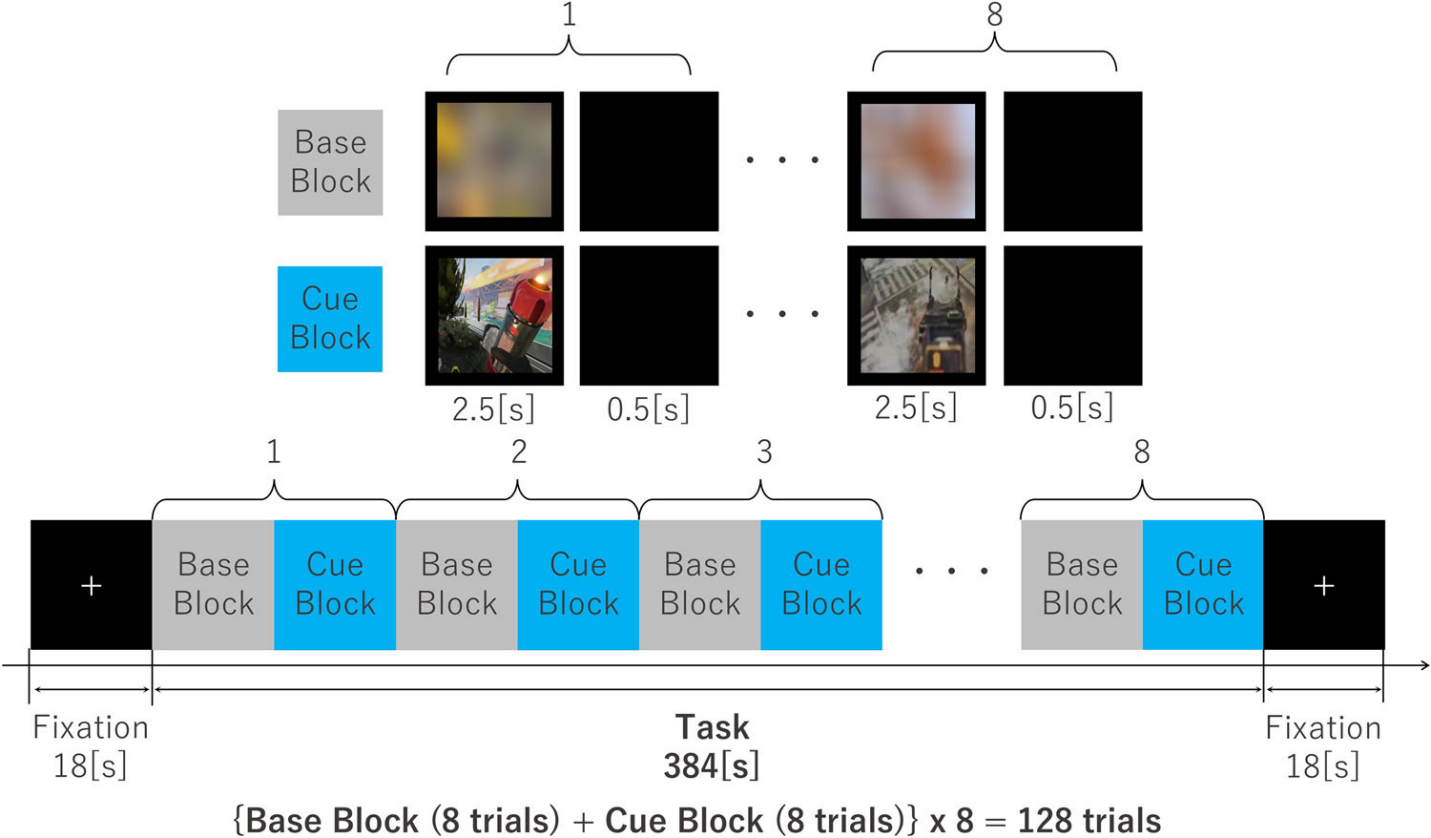
Visual Cue stimulation task. The task consisted of two conditions: a Base and a Cue block. The Base block consisted of eight blurred images, and the Cue block consisted of eight images from the primary game of the individual. The task was performed in pairs, repeated eight times for a total of 384 s.

The visual stimuli were presented in a block design alternating between Base and Cue blocks. Each block contained eight trials. In each trial, each visual stimulus was presented for 2.5 s, followed by a 0.5‐s inter‐stimulus interval (black screen). Thus, each block lasted a total of 24 s. One Base block and one Cue block together formed a block pair, and the full task consisted of 8 such block pairs, totaling 128 trials and 384 s in duration. The task began and ended with an 18‐s fixation cross, serving as a baseline (Figure 1).

The task employed a block design rather than a randomized event‐related design due to technical difficulties in creating the stimulus presentation program. Although stimuli were presented in blocks, neural responses were analyzed using stimulus‐locked epochs, enabling event‐related temporal analysis.

### MEG data acquisition

MEG data were recorded in a magnetically shielded room using a 306‐channel whole‐head magnetoencephalograph (Neuromag Vectorview system, Megin Oy, Espoo, Finland). Data were sampled at 1000 Hz with an online band‐pass filter set to 0.1–200 Hz.

Participants had head position indicator coils (HPIs) attached to three locations on the forehead (center, left, and right) and on both mastoid processes, a total of five locations, and wore nonmagnetic goggles equipped with a reference receiver. Participants sat in a nonmagnetic digitizing chair with a 3D digitizing system transmitter attached to the back, which was connected to the reference receiver via a wire. Positional data for the five HPIs and head shape data, including anatomical landmarks (nasion: NAS, left/right preauricular points: LPA, RPA) and 50–100 additional scalp points along the skull, cheekbones, and nasal bones, were collected using a 3D digitizer.

To monitor eye movement artifacts, four electrodes were attached around the left eye: two above and below for vertical electrooculogram (VEOG), and two on the temples (for horizontal electrooculogram [HEOG]). After electrode attachment, participants removed the nonmagnetic goggles and entered the shielded room, and were seated in the MEG system. The MEG gantry was adjusted to the upright position, and the shielded room was sealed.

Visual stimuli were presented using Presentation software (version 20.1, Neurobehavioral Systems) and projected onto a nonmagnetic screen using a PT‐DW530 projector (Panasonic, 4000 lumens). MEG data acquisition began upon task initiation and terminated immediately after task completion.

In addition to task‐related measurements, MEG empty‐room data were acquired for at least 2 min after the participant completed their task, when the room was vacant, to reduce environmental noise during analysis.

Structural T1‐weighted images were obtained for each participant using a 3D MRI scan to facilitate coregistration of MEG source localization. Imaging parameters were as follows: repetition time (TR) = 8.132 ms; echo time (TE) = 4.2 ms, flip angle = 12°; field of view (FOV) = 230 × 230 mm; matrix = 256 × 256; slice thickness = 1.4 mm; and number of excitations (NEX) = 1. Three anatomical landmarks, the NAS and the LPA/RPA, were marked to serve as reference points for MEG–MRI integration.

### MEG preprocessing and current source estimation

MEG data were first processed using MaxFilter software (Megin Oy), which applies the spatial signal separation algorithm to remove external magnetic noise and correct for head movement. Subsequent preprocessing was conducted using Brainstorm, a MATLAB‐based open‐source platform for MEG/EEG analysis (version 9.11.0 R2021b, MathWorks).^35^

A band‐pass filter (2–30 Hz) was applied to the raw MEG data. Blink artifacts were removed using signal space projections, based on blink events automatically detected through VEOG and HEOG signals. Next, the analysis interval was cut out. Epochs were then extracted and categorized according to stimulus type (Base vs. Cue). Trials exceeding the threshold for physiological noise (gradiometer: >3000 fT, magnetometer: >10,000 fT) were excluded. Remaining trials were averaged per condition (Base and Cue) for each subject to compute visual evoked magnetic fields (VEFs). To ensure a sufficient signal‐to‐noise ratio in VEFs, at least 50 clean trials are typically required.

Structural MRI data were processed using Brainsuite21a to construct individual cortical surface models. These models included labeling of the brain, skull, and scalp tissues and were imported to Brainstorm. The brain model was normalized to the Montreal Neurological Institute (MNI) coordinate system and aligned with the MEG sensor positions using anatomical landmarks (NAS, LPA, and RPA) and 50–100 scalp points collected using a 3D digitizing system. The cortical mesh was resampled to 15,000 vertices per subject for source analysis.

Noise covariance matrices were computed from empty‐room recordings that were band‐pass filtered between 2 and 30 Hz as a preprocessing. These recordings were used to characterize environmental noise and were incorporated into the minimum norm estimation (MNE) source estimation procedure. A forward model was generated using a local sphere‐fitting method based on individual head geometry to model the relationship between cortical sources and MEG sensor signals. Current source estimation was performed using the MNE method applied to condition‐averaged evoked responses for each participant and stimulus condition. Since the sign of the MNE solution can vary based on dipole orientation, absolute values of current amplitudes were used. Source maps were spatially normalized to the ICBM152 Nonlinear Asymmetric brain template and smoothed with a 3‐mm full‐width at half‐maximum (FWHM) Gaussian kernel to reduce local noise and enhance anatomical comparability across participants.

### Group‐level and region of interest–based analyses

VEFs were first inspected using 306‐channel overlay display plots to characterize waveform morphology and identify major response components. Within the IGD–ASD group, Cue versus Base conditions were compared to examine cue‐induced changes. Between‐group comparisons were performed for IGD–ASD‐Cue versus HC‐Cue to investigate IGD–ASD‐specific cue reactivity, and IGD–ASD‐Base versus HC‐Base to assess baseline differences.

Group‐level statistical analyses were conducted on region of interest (ROI)–averaged source amplitudes within predefined time windows (40–130, 130–200, and 200–270 ms) derived from VEF morphology. Two‐tailed nonparametric permutation tests were used for within‐group and between‐group comparisons. False discovery rate (FDR) correction (Benjamini–Hochberg) was applied across ROIs and time points within each comparison to control for multiple testing. Source estimates were averaged within each group and condition for statistical analysis.

According to prior literature and regions showing prominent source activity, 20 ROIs were defined for the analysis. These included the left and right hemispheres of the fusiform gyrus,^6^ inferior parietal lobule,^33^ insular cortex,^36^ lateral occipital gyrus,^37^ lingual gyrus,^38^ parahippocampal gyrus,^39^ cingulate gyrus,^40^ orbitofrontal cortex,^41^ frontal gyrus,^6^ and temporal gyrus,^37^ resulting in a total of 20 regions. The shape analysis and anatomical labeling of these regions were conducted using the Desikan–Killiany atlas^42^ within Brainstorm (Figure S1).

We then conducted a Spearman's rank correlation analysis between activity in the relevant ROIs identified in the above‐mentioned analyses and the behavioral measures, including gaming hours per week, IAT, CBCL total score, and CBCL subscale reflecting the typical features of ASD. The correlation coefficients are provided, with a significance level of *p* < 0.05.

## RESULTS

### Characteristics of participants

We found no significant differences in the mean age and alternative IQ between the IGD–ASD and HC groups (Table 1). The IAT scores exhibited a significant difference between groups (*p =* 0.00086), with the IGD–ASD group scoring significantly higher (56.27 ± 16.54) than the HC group (34.53 ± 9.44). The CBCL scores were significantly different between groups (*p =* 0.00000152), with the IGD–ASD group scoring higher (54.73 ± 24.46) than the HC group (8.85 ± 6.69).

**Table 1:** Characteristics of participants.

	IGD–ASD (*n* = 11)	HC (*n* = 13)	*p*
Age (years)	15.27 ± 2.24	15.69 ± 1.80	0.54
IQ (JART)	99.09 ± 9.47	101.54 ± 6.29	0.49
Gaming hours/week	69.27 ± 30.6	15.92 ± 13.79	0.00018*
IAT total	56.27 ± 17.35	34.53 ± 9.83	0.0025*
CBCL total	54.73 ± 24.46	8.85 ± 6.69	0.000080*
CBCL ASD (Withdrawn and PDP)	7.82 ± 4.42	1.77 ± 1.69	0.00066*

*Note*: Data are presented as means ± standard deviation.

Abbreviations: ASD, autism spectrum disorder; CBCL, Child Behavior Checklist; HC, healthy control; IAT, Japanese version of Young's Internet Addiction Test; IGD, internet gaming disorder; IQ, intelligence quotient; JART, Japanese Adult Reading Test; PDP, pervasive developmental problems.

* Significant at *p* < 0.05 (Mann–Whitney *U* test).

### Neural activity measured by VEFs

**Figure 2:**
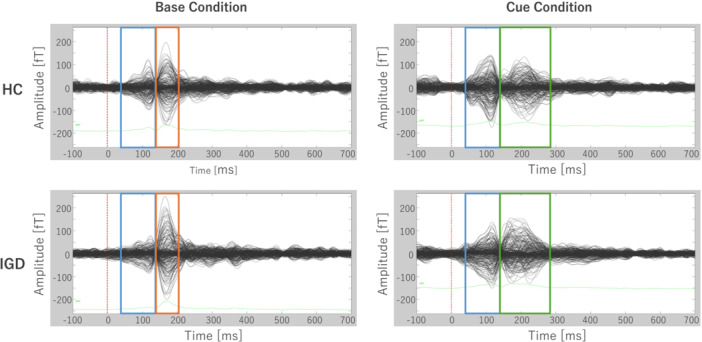
306‐Channel superimposed display of the average stimulus‐evoked magnetic fields for the internet gaming disorder (IGD)–autism spectrum disorder (ASD) and healthy control (HC) groups, and under base and cue conditions. The visual evoked magnetic fields (VEFs) obtained for each of the four conditions (red dotted line: the starting point of stimulus image presentation). HC group Base condition, HC group Cue condition, IGD group Base condition, and IGD group Cue condition. The first‐wave response was observed approximately 40–130 ms in both conditions (blue box), the second‐wave response approximately 130–200 ms in the Base condition (orange box), and the second‐wave response approximately 130–270 ms in the Cue condition (green box).

The averaged VEFs, expressed as magnetic field amplitude in femtotesla over time in milliseconds, were obtained for each group and stimulus condition: HC‐Base, HC‐Cue, IGD–ASD‐Base, and IGD–ASD‐Cue (Figure 2). A prominent initial waveform response was observed between 40 and 130 ms in both groups and stimulus conditions (blue box). In the Base stimulus condition for both groups, a second waveform response was observed between 130 and 200 ms (orange box). A more prolonged second waveform response was observed in the Cue‐stimulus condition for both groups within 130–270 ms (green box). Based on these responses, we conducted group comparisons within three predefined time windows: 40–130, 130–200, and 200–270 ms. Source‐level neural activity was estimated for the 40–270 ms time window based on VEF responses. Group averages for each condition and time window are visualized in inferior views of cortical activation (Figure S2a–c).

### Group comparisons of significant neural activity among ROIs

Within the IGD–ASD group, based on nonparametric permutation tests with FDR correction, we found a significant difference between the Base and Cue stimuli at 137 ms in the right fusiform gyrus (*p* = 0.000039), with neural activity higher under Cue stimuli than under Base stimuli (Table 2).

**Table 2:** Comparison in the internet gaming disorder (IGD)–autism spectrum disorder (ASD) group between Cue and Base conditions at a significant region of interest (ROI) within 130–200 ms.

ROI	Time (ms)	*X*	*Y*	*Z*	Current source estimate		*p*
					Base (E−11*Am)	Cue (E−11*Am)	
Right fusiform	137	39.2	−49.5	−23.8	1.02 ± 0.33	1.78 ± 0.63	0.000039*

Abbreviation: E−11*Am, multiplied by ten to the power of negative eleven (10^−11^ × Am).

* Significant at *p* < 0.00014 (after false discovery rate [FDR] correction).

**Figure 3:**
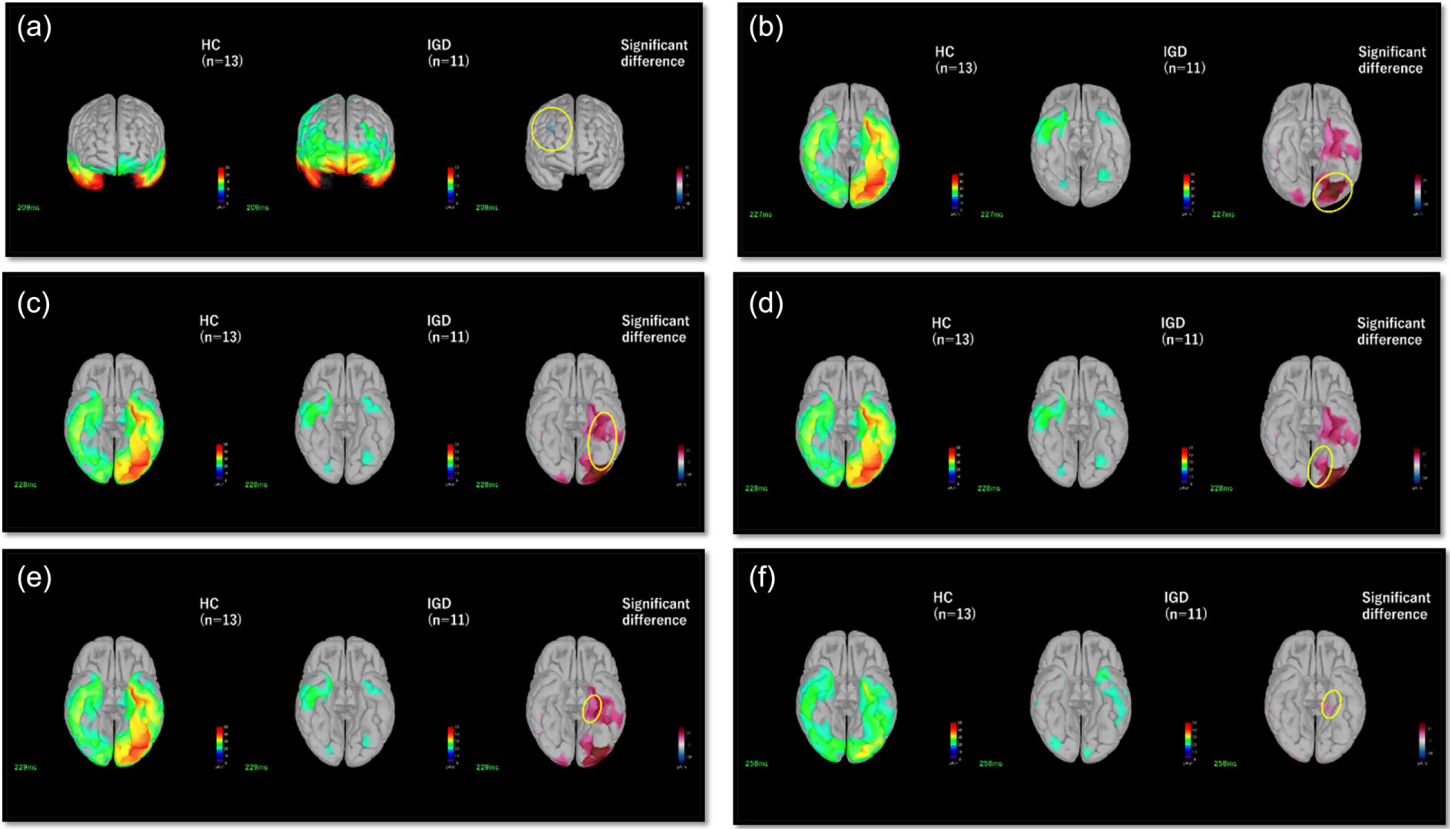
Comparison of the current source estimate between internet gaming disorder (IGD) and healthy control (HC) under cue condition at significant regions of interest (ROIs) within 200–270 ms. In the display of differences, blue indicates significantly stronger activity in the IGD group, and red indicates significantly stronger activity in the HC group. The significance level after false discovery rate (FDR0 correction was 0.0033. (a) Right frontal at 209 ms. (b) Left lateral occipital at 227 ms. (c) Left fusiform at 228 ms. (d) Left lingual at 228 ms. (e) Left parahippocampal at 229 ms. (f) Left parahippocampal at 258 ms.

In the Cue‐stimuli condition, we found significant differences between IGD–ASD and HC within the 200–270 ms time window across six ROIs (Table 3). The IGD–ASD group showed a significantly higher neural activity in the right frontal region (*p* = 0.0028) than the HC group. Moreover, the IGD–ASD group showed a significantly lower neural activity in the left lateral occipital (*p* = 0.000092), left fusiform (*p* = 0.00025), left lingual (*p* = 0.0017), and left parahippocampal (twice, around 228 ms and around 258 ms) (*p* = 0.000049 and 0.0027) regions compared with the HC group (Figure 3).

**Table 3:** Comparison between internet gaming disorder (IGD)–autism spectrum disorder (ASD) and healthy control (HC) under cue condition at significant regions of interest (ROIs) within 200–270 ms.

ROIs	Time (ms)	*X*	*Y*	*Z*	Current source estimate		*p*
					HC (E−11*Am)	IGD (E−11*Am)	
Right frontal	209	30.1	39.4	28.8	0.52 ± 0.11	0.79 ± 0.26	0.0028*
Left lateral occipital	227	−28.2	−89.9	−20.8	2.49 ± 1.17	1.09 ± 0.41	0.000092*
Left fusiform	228	−31.7	−30.9	−21.0	3.36 ± 1.31	1.58 ± 0.55	0.00025*
Left lingual	228	−21.6	−83.9	−17.6	2.75 ± 1.07	1.28 ± 0.81	0.0017*
Left parahippocampal	229	−23.3	−35.7	−19.3	2.64 ± 1.08	1.03 ± 0.29	0.000049*
Left parahippocampal	258	−23.3	−35.7	−19.3	2.24 ± 1.02	1.21 ± 0.37	0.0027*

Abbreviation: E−11*Am, multiplied by ten to the power of negative eleven (10^−11^ × Am).

* Significant at *p* < 0.0033 (after false discovery rate [FDR] correction).

Under the Base stimulus condition, we found significant differences between IGD–ASD and HC within the 130–200 ms window in two ROIs (Table 4). The IGD–ASD group showed a significantly lower neural activity in the right fusiform (*p* = 0.00021) and right parahippocampal regions (*p* = 0.000012), respectively.

**Table 4:** Comparison between internet gaming disorder (IGD)–autism spectrum disorder (ASD) and healthy control (HC) under base condition at significant regions of interest (ROIs) within 130–200 ms.

ROI	Time (ms)	*X*	*Y*	*Z*	Current source estimate		*p*
					HC (E−11*Am)	IGD (E−11*Am)	
Right fusiform	135	29.0	−28.9	−28.1	1.86 ± 0.79	1.29 ± 0.52	0.00021*
Right parahippocampal	136	29.0	−28.9	−28.1	1.35 ± 0.90	0.96 ± 0.50	0.000012*

Abbreviation: E−11*Am, multiplied by ten to the power of negative eleven (10^−11^ × Am).

* Significant at *p* < 0.00021 (after false discovery rate [FDR] correction).

Significant effects represent peak latency points within the predefined time windows, reflecting transient event‐related components rather than sustained activation across the entire window. The time points shown in Tables 2, 3, 4 indicate the peak time points of each event‐related component for which significant differences were found after FDR correction in each ROI; other event‐related components did not show significant differences after correction.

### Correlation of neural activity and clinical assessments

The Spearman's rank correlation analysis revealed some significant associations between neural activity in the relevant ROIs and behavioral measures (Table 5). The number of gaming hours per week was associated with neural activity in the right frontal, left lateral occipital, and left parahippocampal regions under Cue stimuli. The IAT was associated with neural activity in the left parahippocampal region under the Cue stimulus at 229 ms. The CBCL total and CBCL subscale related to ASD were associated with the neural activity of right frontal, left lateral occipital, left fusiform, left lingual, and left parahippocampal under Cue stimuli.

**Table 5:** Spearman's rank correlations between significant regions of interest (ROIs) activity between two groups and behavioral measures (pooled sample, *N* = 24).

ROIs	Condition	Time (ms)	Gaming hours/week	IAT	CBCL total	CBCL ASD (Withdrawn and PDP)
Right frontal	Cue	209	0.405*	0.326	0.594**	0.424*
Left lateral occipital	Cue	227	−0.497*	−0.290	−0.628**	−0.568**
Left fusiform	Cue	228	−0.319	−0.361	−0.620**	−0.628**
Left lingual	Cue	228	−0.365	−0.184	−0.546**	−0.454*
Left parahippocampal	Cue	229	−0.441*	−0.407*	−0.638**	−0.661**
Left parahippocampal	Cue	258	−0.525**	−0.389	−0.428*	−0.383
Right fusiform	Base	135	−0.177	−0.236	−0.315	−0.267
Right parahippocampal	Base	136	−0.184	−0.226	−0.015	−0.035

*Note*: Correlation coefficients were provided between ROI and behavioral measures.

Abbreviations: ASD, autism spectrum disorder; CBCL, Child Behavior Checklist; IAT, Japanese version of Young's Internet Addiction Test; PDP, pervasive developmental problems.

* Significant at *p* < 0.05.

** Significant at *p* < 0.01.

## DISCUSSION

We examined cortical neural responses to gaming cues in individuals with IGD comorbid with ASD using MEG. Rather than replicating the typical reward‐circuit hyperactivation reported in IGD alone, this comorbid group showed increased right frontal activity and reduced activation in left occipital, fusiform, lingual, and parahippocampal regions. These differences emerged within 137–230 ms after stimulus onset, earlier than the LPP window typically associated with reward‐driven craving processes, which generally occurs around 300–700 ms after stimulus onset.^8^ Because activity in this time window was limited in the 306‐channel overlay display, a comparison of the time window corresponding to LPP between groups was not performed in this study. Issues due to differences in tools, such as differences in the detectable current direction between EEG and MEG, are hypothesized. On the other hand, comparing weekly game play time also suggests demographic differences between the IGD group in previous studies and the IGD–ASD group in this study, and differences in the underlying mechanisms between the pure IGD and IGD–ASD groups cannot be ruled out.

The higher right fusiform activation observed within the IGD–ASD group at 137 ms during the Cue, compared with the Base condition (Table 2), may reflect enhanced visual salience and top‐down attentional control toward gaming‐related stimuli.^20^ Although the right fusiform gyrus is not typically considered part of the craving pathway in IGD, this early activation may instead represent the restricted interests and domain‐specific expertise often seen in ASD.^43^ When exposed to their preferred gaming stimuli, ASD individuals may show robust perceptual engagement. When these behaviors meet the provisional criteria for IGD in the DSM‐5‐TR, they may manifest as comorbid excessive gaming and restricted interest.

Between‐group comparisons under the Cue condition revealed significantly greater activation in the right frontal region at 209 ms, and lower activation in the left lateral occipital (227 ms), left fusiform (228 ms), left lingual (228 ms), and left parahippocampal regions (229 and 258 ms) in the IGD–ASD group compared with HCs (Table 3). An fMRI study^44^ demonstrated that patients with persistent IGD exhibited greater activation in the right DLPFC during a game‐related craving cue task than those who had recovered from IGD, which they interpreted as a strong attentional bias toward game‐related cues. Our current findings, although obtained with different imaging tools and time windows, are similar. Hyperactivation of the frontal lobe by craving‐related visual stimuli has been repeatedly observed in addictions other than internet gaming, such as alcoholism,^45^ smoking,^46^ and gambling,^47^ although the imaging tools, analytical methods, and time periods in which the findings appear vary. In our findings, the frontal P2m component of the stimulus‐evoked magnetic field evoked by visual stimuli showed a significant positive correlation with gaming hours per week, total CBCL score, and CBCL‐related ASD score (Table 5). The frontal P2m component of the stimulus‐evoked magnetic field evoked by visual stimuli is thought to reflect conscious perception,^48^ suggesting that it may be involved in craving via conscious perception of the desired target. By contrast, IGD patients repeatedly demonstrated decreased right DLPFC activity during resting states^49^ and when evoked by visual stimuli unrelated to internet games^50^, ^51^ suggesting that they were unable to maintain attentional allocation to anything other than the internet games they were craving. Furthermore, in exceptional circumstances, IGD patients also demonstrated decreased right DLPFC activity when exposed to craving‐related stress.^37^ Further research is needed to clarify the detailed mechanisms underlying the changes in activity across situations. Although ASD has been associated with atypical reward processing, reward responsivity in ASD is often domain‐specific. In IGD–ASD, gaming cues may be processed primarily as restricted‐interest stimuli rather than purely addictive rewards, potentially explaining the relative reduction of classic reward‐circuit signatures.

The ventral visual pathway,^52^ extending from the lateral occipital cortex to the fusiform gyrus and parahippocampal gyrus, is considered a specific brain region that responds preferentially to specific categories of visual objects. In particular, the fusiform gyrus mediates face‐specific recognition memory, the parahippocampal gyrus mediates scene‐specific recognition memory, and the lateral occipital cortex transmits category‐general recognition memory neural codes. Patients with ASD have been shown to exhibit greater occipital cortical activation^53^ in response to unexpected visual change stimuli than controls. By contrast, patients with IGD have reduced resting regional homogeneity (ReHo)^54^ in the occipital lobe compared with healthy individuals, and patients with online gaming addiction (POGA) have reduced gray matter volume^55^ in the left inferior occipital gyrus compared with HCs. The fusiform face area has been implicated in face recognition, but fMRI has shown insufficient activation of the fusiform^56^ gyrus during face processing in patients with ASD. Whereas fMRI showed that, in subjects with ASD, viewing images related to limited personal interests and controls viewing images related to strong interests or hobbies, images related to interests and expertise evoked stronger FFA responses.^43^ These findings suggest that the reactivity of brain regions involved in social functioning is not inherently reduced in ASD patients, but may instead be activated by different environmental stimuli. However, a study conducted MRI DKI on patients with internet gaming addiction (IGA), and HCs found that the mean kurtosis metrics in the left fusiform gyrus was lower^57^ in the IGA group than in the control group, suggesting that those in the IGA group may be damaged by harmful visual stimuli resulting from excessive exposure to visual stimuli while gaming online. The parahippocampal gyrus, which is thought to be involved in scene recognition, has been shown to exhibit increased activity in the left hemisphere^58^ and decreased activity in the right hemisphere during task performance in ASD. By contrast, hypoactivity in the left parahippocampal gyrus^59^, ^60^ during task performance has been reported in IGD. The dorsal visual pathway,^61^ including the lateral occipital cortex and lingual gyrus, is thought to be involved in object localization and interaction. Although strong task‐related activation^58^ of the lingual gyrus and abnormal resting‐state connectivity (both on the right side) have been reported in ASD patients,^62^ abnormalities in the left lingual gyrus have not been noted in these large‐scale studies. However, patients with IGD showed hypoactivity in the left lingual gyrus^38^, ^60^ during task performance compared with controls. Combining the findings of these previous studies with the results of this study, we found that areas of the left ventral and dorsal visual pathways showed hyperactivation in response to visual stimuli in patients with ASD alone, but hypoactivation or volume reduction in patients with IGD–ASD. These areas may be damaged by harmful visual stimuli^55^, ^57^ in patients with IGD. However, to confirm this, comparative studies are needed, such as those before and after the onset of IGD in ASD and those before and after IGD treatment in patients with ASD–IGD.^17^, ^20^, ^63^

Under the Base condition, the IGD–ASD group also showed reduced activation in the right fusiform (135 ms) and parahippocampal (136 ms) regions compared with controls (Table 4).^8^ However, we found no correlation of these brain areas under Base conditions with any CBCL scores. Previous fMRI studies have shown that ASD patients exhibit higher activation in the bilateral fusiform gyrus^43^ compared with controls in response to personally relevant low‐interest stimuli,^45^ but significantly lower activation in the bilateral fusiform gyrus compared with controls in response to other visual stimuli.^56^ Previous MEG studies have also shown lower activation in the right fusiform gyrus in response to visual stimuli at approximately 150 ms in patients with ASD.^64^ In patients with IGD, MRI DKI revealed decreased radial kurtosis (K⊥) in the right fusiform gyrus.^57^ A meta‐analysis of fMRI in ASD has shown lower activation in the right parahippocampal gyrus. In IGD patients, fMRI during a card‐guessing task demonstrated reduced activation in the right parahippocampal gyrus.^59^ These results, common to this study and previous studies, suggest that the ventral visual pathway is hypoactive in response to non‐preferred stimuli in both ASD and IGD.^65^

The observed pattern likely reflects the interaction of both conditions. Early fusiform hyperactivity aligns with literature on restricted‐interest processing in ASD, whereas hypoactivation of ventral visual regions (fusiform, lingual, and parahippocampal) has been reported in IGD. The right frontal P2m effect may represent top‐down cognitive control or salience processing influenced by both ASD‐related perceptual specialization and addiction‐related attentional bias.

These findings emphasize the need to differentiate between addictive mechanisms and ASD‐specific features underlying excessive gaming, as suggested by a previous study.^16^ Furthermore, the millisecond‐level precision of MEG may serve as a promising tool for identifying neurophysiological biomarkers to guide individualized prevention and treatment strategies in neurodiverse populations.

This study has several limitations, including a small, all‐male sample, a cross‐sectional design, and the use of static images rather than dynamic gaming stimuli. The study also focused exclusively on individuals addicted to first‐person shooter and third‐person shooter games, and participants played specific games, introducing uncontrolled variability. Individuals with severe IGD were excluded, limiting the generalizability of our findings to the full clinical spectrum. Moreover, we compared only IGD–ASD individuals with HCs. The lack of ASD‐only and IGD‐only comparison groups limits condition‐specific interpretation of the findings. Because this study was conducted in the context of clinical practice for the treatment of IGD, recruitment challenges required focusing on the comorbid IGD–ASD phenotype. Thus, future studies including ASD‐only and IGD‐only groups are needed to clarify overlapping and distinct brain activation patterns. The short stimulus duration may also have limited the detection of longer‐lasting neural activity typically observed in fMRI studies. These factors may reduce generalizability and ecological validity. Future research should include larger, more diverse samples, incorporate behavioral indices of craving and executive control, and employ longitudinal, multimodal designs combining MEG and fMRI to elucidate further the structural–functional mechanisms underlying IGD–ASD comorbidity.

In conclusion, individuals with IGD comorbid with ASD exhibited distinctive cortical activation patterns, characterized by increased right frontal activity and decreased left occipital, fusiform, lingual, and parahippocampal activation during exposure to gaming cues. Although these findings highlight atypical visual and cognitive processing mechanisms of this comorbid group, future studies may clarify the pathology of these significant populations by comparing individuals with ASD and those with ASD–IGD, as well as by examining the neurophysiological characteristics of individuals with ASD who develop or recover from IGD.

## AUTHOR CONTRIBUTIONS

**Faisal Budisasmita Paturungi Parawansa**: Formal analysis; investigation; writing—original draft; visualization. **Atsunori Sugimoto**: Conceptualization; methodology; formal analysis; investigation; resources; writing—original draft. **Ekachaeryanti Zain**: Conceptualization; methodology; formal analysis; investigation; writing—original draft; visualization. **Yukina Nakazawa**: Software; formal analysis; investigation; data curation; writing—review and editing. **Fuuta Sakuma**: Software; formal analysis; investigation; data curation; writing—review and editing. **Kiyohiro Yoshinaga**: Conceptualization; methodology; investigation; resources; writing—review and editing; project administration; funding acquisition. **Muhammad Dwi Wahyu**: Investigation; writing—review and editing. **Hiroyuki Kasahara**: Investigation; writing—review and editing. **Jun Egawa**: Conceptualization; supervision; writing—review and editing. **Hiroshi Shirozu**: Resources; writing—review and editing. **Atsuhiko Iijima**: Supervision; writing—review and editing. **Shuken Boku**: Supervision; writing—review and editing. All authors contributed to and have approved the final manuscript.

## CONFLICT OF INTEREST STATEMENT

A.S. is a director of Medica Staff Promotion Co., Ltd. and representative of F.I.W. LLC. A.S. has received lecture fees, manuscript royalties, and other remuneration from the following companies: Springer Nature Switzerland, Takeda Pharmaceutical Company Limited, Nobelpharma Co., Ltd., Jiho Inc., Shinkoh Igaku Shuppan Co., Ltd., Sentan Igaku‐Sha Ltd., Medibanks Co., Ltd., and Television Niigata Network Co., Ltd. The other authors have no conflicts of interest to declare.

## ETHICS APPROVAL STATEMENT

This study was approved by the Ethical Review Committee of Niigata University (approval number: 2020‐0260) and the ethics committees of the Niigata Prefectural Psychiatric Center. All procedures involving human participants were conducted in accordance with the ethical standards outlined in the Declaration of Helsinki and relevant institutional ethical guidelines.

## PATIENT CONSENT STATEMENT

Written informed consent was obtained from all participants and, for minors, from their legal guardians, after a full explanation of the study.

## CLINICAL TRIAL REGISTRATION

N/A.

## Supporting information

Supporting Information.
