# Supplementary material for: Atypical cortical neural activity in internet gaming disorder comorbid with autism spectrum disorder during a cue reactivity task: A magnetoencephalography study
Source: PCN Rep. 2026 Mar 16;5(1):e70312. doi: 10.1002/pcn5.70312 (PMC13097378; doi:10.1002/pcn5.70312)
Supplement: Supplementary file 1 — Supporting Information. [file PCN5-5-e70312-s001.docx]

**SUPPLEMENTARY FIGURE’S**


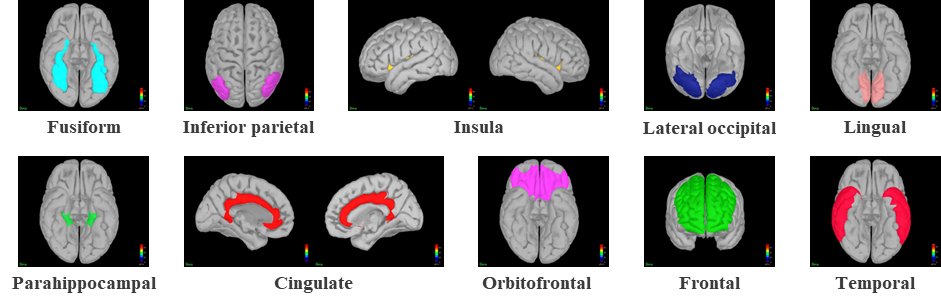


**Supplementary Figure. 1** Regions defined as ROIs


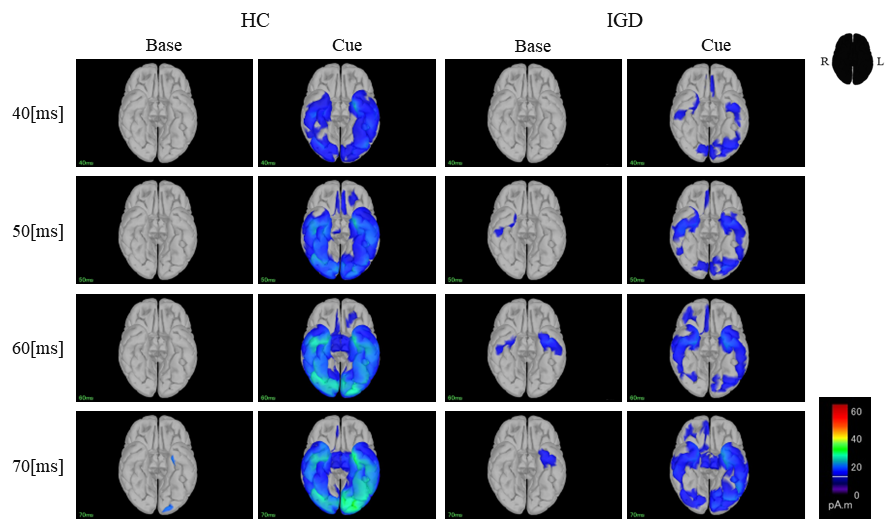


**Supplementary Figure. 2a** Results of current source estimation from 40 to 270 ms (1)


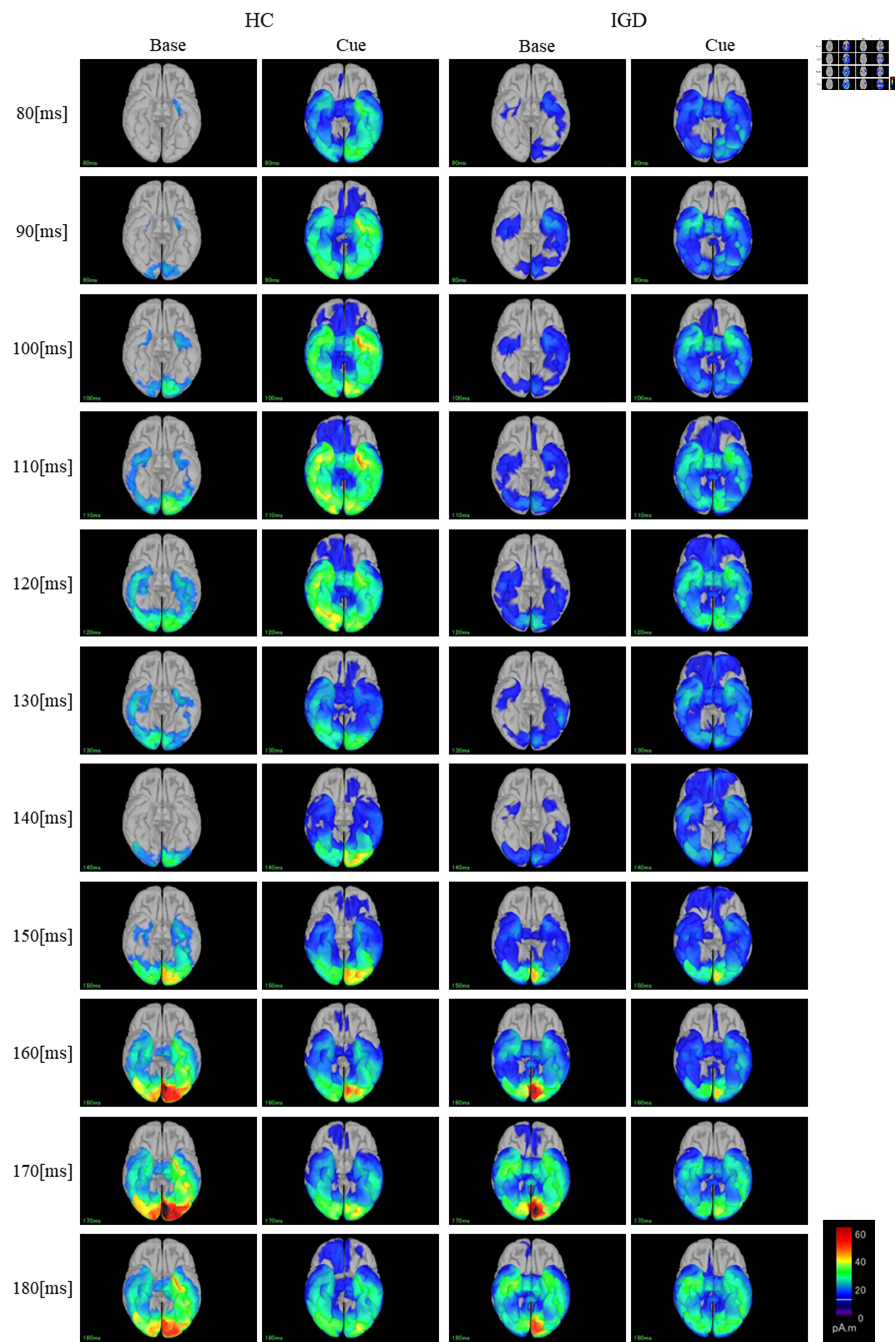


**Supplementary Figure. 2b** Results of current source estimation from 40 to 270 ms (2)


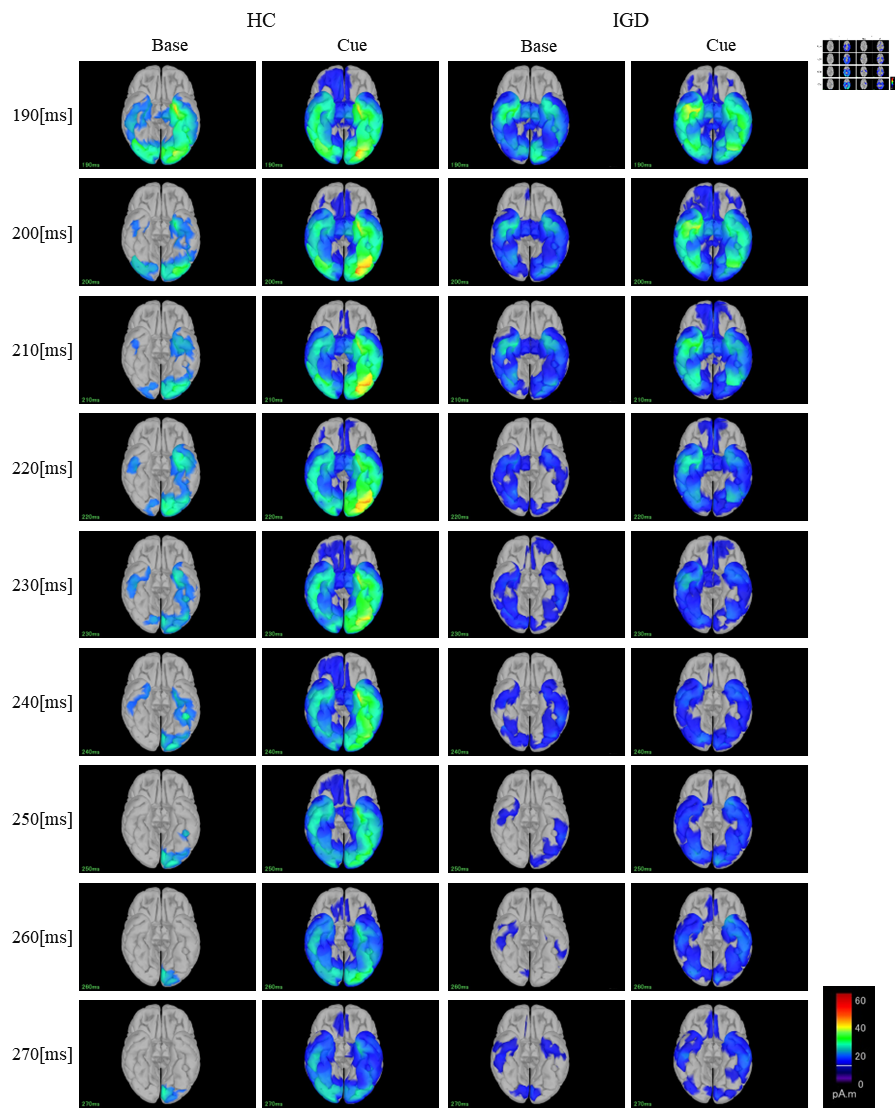


**Supplementary Figure. 2c** Results of current source estimation from 40 to 270 ms (3)
